# Combinations of *Beauveria bassiana* and spinetoram for the management of four important stored-product pests: laboratory and field trials

**DOI:** 10.1007/s11356-022-23753-8

**Published:** 2022-11-16

**Authors:** Waqas Wakil, Nickolas G. Kavallieratos, Erifili P. Nika, Mirza Abdul Qayyum, Taha Yaseen, Muhammad Usman Ghazanfar, Muhammad Yasin

**Affiliations:** 1 Department of Entomology, University of Agriculture, Faisalabad, 38040 Pakistan; 2 Senckenberg German Entomological Institute, Eberswalder str. 90, 15374 Müncheberg, Germany; 3 Laboratory of Agricultural Zoology and Entomology, Department of Crop Science, Agricultural University of Athens, 75 Iera Odos str, 11855 Athens, Attica Greece; 4 Institute of Plant Protection, Muhammad Nawaz Shareef (MNS) University of Agriculture, Multan, 60000 Pakistan; 5 Department of Plant Pathology, College of Agriculture, University of Sargodha, Sargodha, 40100 Pakistan; 6 Department of Entomology, Faculty of Agriculture and Environment, The Islamia University of Bahawalpur, Bahawalpur, 63100 Pakistan

**Keywords:** Biological control, Spinosyns, Stored-product insects, Mortality, Progeny, Persistence, Field experiments

## Abstract

The current study examines the efficacy of the semi-synthetic insecticide spinetoram and entomopathogenic fungi *Beauveria bassiana* (Balsamo-Crivelli) Vuillemin (Hypocreales: Cordycipitaceae) as wheat protectants against the lesser grain borer, *Rhyzopertha dominica* (F.) (Coleoptera: Bostrychidae), the red flour beetle, *Tribolium castaneum* (Herbst) (Coleoptera: Tenebrionidae), the granary weevil, *Sitophilus granarius* (L.) (Coleoptera: Curculionidae), and the khapra beetle, *Trogoderma granarium* Everts (Coleoptera: Dermestidae), under laboratory and field trials. One dose of *B. bassiana*, i.e., 1 × 10^7^ conidia/kg wheat, two doses of spinetoram, i.e., spine1: 0.05 ppm (mg/kg wheat), spine2: 0.1 ppm, and their combinations (Bb + spine1, Bb + spine2) were evaluated at 20, 25, and 30 °C. All treatments provided significantly higher mortality at 30 °C compared with the other two temperatures. Maximum mortality levels were observed in the treatments where *B. bassiana* was combined with the higher dose of spinetoram (0.1 ppm). All treatments reduced progeny production in comparison with the control groups. Maximum progeny reduction was observed at 30 °C, on wheat treated with the Bb + spine2 combination. The combination Bb + spine2 also provided elevated mortality rates in both laboratory and field persistence trials, but at 180 days caused moderate mortality to all tested insect species. Concerning progeny, at laboratory persistence trials, the combination Bb + spine2 exhibited the lowest offspring emergence to all tested species compared to the other treatments and control. Overall, our study showed that *R. dominica* was the most susceptible species followed by *S. granarius*, *T. castaneum*, and *T. granarium*. Our findings revealed that the combination of *B. bassiana* and spinetoram may be a useful tool for efficient and advanced integrated pest management strategies for long storage periods under multiple temperatures.

## Introduction

Throughout the world, 20–40% of the crops is lost due to diseases and pests (CABI 2022). More than 1000 species of pests, belonging mainly to Coleoptera, Lepidoptera, and Acarina, are responsible for the quantitative and qualitative degradation of stored commodities (Rajendran and Sriranjini 2008). The lesser grain borer, *Rhyzopertha dominica* (F.) (Coleoptera: Bostrychidae), is an important primary pest, infesting 115 different products (Hill 2003; Hagstrum and Subramanyam 2009). It is frequently found at mills, local markets, grain elevators, stables, storages, and food stores (Hagstrum and Subramanyam 2009; Hagstrum et al. 2013). Interestingly, this species infests ripe cereals prior to harvest (Hill 2003). The red flour beetle, *Tribolium castaneum* (Herbst) (Coleoptera: Tenebrionidae), is a secondary stored-product insect pest of high economic importance infesting 246 commodities, but milled products like flours are preferable for its development (Hill 2003; Rees 2004; Hagstrum and Subramanyam 2009). It usually occurs in retail stores, storages, mills, bakeries, and henhouses (Rees 2004; Hagstrum et al. 2013). The granary weevil, *Sitophilus granarius* (L.) (Coleoptera: Curculionidae), is another primary pest occurring in storages, pet stores, stables, and grain bins (Hagstrum and Subramanyam 2009). This pest is of major importance, particularly in temperate climatic regions, and it has been documented to infest 53 commodities (Rees 2004; Hagstrum and Subramanyam 2009; Kumar 2017). Also, the khapra beetle, *Trogoderma granarium* Everts (Coleoptera: Dermestidae) is one of the most dangerous primary pests of stored-products (Hill 2003; Rees 2004), infesting 96 commodities worldwide (Hagstrum et al. 2013). Warehouses, mills, breweries, and grain elevators are its common habitats (Hagstrum and Subramanyam 2009; Hagstrum et al. 2013).

Management of the aforementioned stored-product insect pests is feasible through the application of insecticides as grain protectants (Kavallieratos et al. 2020a, b, 2021a, b; Wakil et al. 2021a, 2022). Among different types of insecticides used as grain protectants, pyrethroids, organophosphates, and carbamates have been extensively used in stored-product protection (Attia et al. 2020). Previous studies have documented their long-term residual activity against a wide variety of stored-product pests (Wakil et al. 2012, 2021b; Wakil and Schmitt 2015; Scheff et al. 2020; Morrison et al. 2021). However, the constant and extended use of several insecticidal active ingredients has led to the emergence of resistance to target insect species, and consequently to insufficient management (Attia et al. 2017; Yao et al. 2019; Khaliq et al. 2020; Cui et al. 2021). Therefore, the need for new and alternative insecticides is intense and crucial.

One other way to manage stored-product pests is linked with the utilization of living organisms like parasitoids, pathogens, predators, viruses, protozoa, bacteria, and fungi (Flinn and Schöller 2012; Schöller et al. 2018). Biological control, unlike common chemical insecticides, has no harmful residues (Flinn and Schöller 2012). Among biological control agents, entomopathogenic fungi exhibit high scientific interest due to their broad spectrum of hosts (Batta and Kavallieratos 2018; Wakefield 2018). Their mode of action against insects is based on six steps beginning with the percutaneous attachment of the spore, the germination on the host cuticle, the deep penetration inside the host, the weakening of the host immune defense system, the proliferation of the fungi inside the host, and lastly the new conidia emerge from the dead insect (Zimmermann 2007). Previous studies have documented the elevated activity of entomopathogenic fungi against numerous stored-product insect pests (Athanassiou et al. 2008a; Shafighi et al. 2014; Batta and Kavallieratos 2018; Mohammed et al. 2019; Wakil et al. 2021a, b, c, d, 2022). More specifically, different entomopathogenic fungi isolates of *Beauveria bassiana* (Balsamo-Crivelli) Vuillemin (Hypocreales: Cordycipitaceae), *Metarhizium anisopliae* (Metschnikoff) Sorokin (Hypocreales: Clavipitaceae), *Lecanicillium attenuatum* (Petch) Zare and Gams (Hypocreales: Cordycipitaceae), *Isaria fumosorosea* (Wize) (Hypocreales: Clavicipitaceae), and *Chaetomium globosum* (Kunze) (Sordariales: Chaetomiaceae) have been tested as grain protectants against adults of *R. dominica*, *S. granarius*, *T. granarium*, and *T. castaneum*, the saw-toothed grain beetle, *Oryzaephilus surinamensis* (L.) (Coleoptera: Silvanidae), the rusty grain beetle, *Cryptolestes ferrugineus* (Stephens) (Coleoptera: Laemophloeidae), the rice weevil, *Sitophilus oryzae* (L.) (Coleoptera: Curculionidae), and the psocid *Liposcelis paeta* (Pearman) (Psocoptera: Liposcelididae) (Athanassiou et al. 2007, 2008a, b; Shafighi et al. 2014; Saeed et al. 2020; Wakil et al. 2021a, b, c, d). In order to enhance the insecticidal properties of entomopathogenic fungi, they can be combined with chemical insecticides, natural enemies, diatomaceous earths (DEs), and natural products (Batta and Kavallieratos 2018).

Spinetoram is a semi-synthetic insecticide belonging to the spinosyn family, i.e., secondary metabolites of the Gram-positive, aerobic, and soil bacterium *Saccharopolyspora spinosa* Mertz and Yao (Pseudonocardiales: Pseudonocardiaceae) (Dripps et al. 2011). It is a combination of 3′-*O*-ethyl-5,6-dihydro spinosyn J and 3′-*O*-ethyl spinosyn L (Dripps et al. 2011). Spinetoram has been previously tested against stored-product insect pests. For instance, when spinetoram was applied on concrete, it was the most effective insecticide among imidacloprid and thiamethoxam against adults and young larvae of the confused flour beetle, *Tribolium confusum* Jacquelin du Val (Coleoptera: Tenebrionidae) (Saglam et al. 2013). Later, Vassilakos and Athanassiou (2015) documented the residual insecticidal properties of spinetoram against *S. oryzae*, *T. confusum*, and *O. surinamensis*, on galvanized steel and concrete for a period of 6 months. The results were promising as this insecticide exhibited persistence, remained stable, and caused elevated mortality levels to the tested species. According to Vassilakos et al. (2012) and Rumbos et al. (2018), spinetoram is an effective grain protectant against *S. granarius*, *S. oryzae*, *O. surinamensis*, *T. castaneum*, *T. confusum*, *R. dominica*, *C. ferrugineus*, and the larger grain borer, *Prostephanus truncatus* (Horn) (Coleoptera: Bostrychidae). Interestingly, spinetoram has strong residual efficacy against *S. oryzae*, *R. dominica*, and *T. confusum* for a period of 8 months on stored wheat, lacking considerable degradation (Vassilakos et al. 2015). Similarly, Ksoura et al. (2021) found that this compound was stable for a 5-month period, killing all exposed *R. dominica* and *S. oryzae* adults, after the application of 10 ppm on wheat and maize. It should be noted that spinetoram impacts the progeny production of stored-product insect pests. For example, 1 ppm was enough to suppress the emergence of *R. dominica* offspring (Ksoura et al. 2021). For *P. truncatus*, 0.5-ppm spinetoram resulted to complete inhibition of progeny production (Vassilakos et al. 2012).

After a meticulous search of the literature, there is no published information about the efficacy of *B. bassiana* in combination with spinetoram as grain protectants against stored-product insect pests. Therefore, the objective of the current study was to shed light on the efficacy of *B. bassiana* and spinetoram, alone or in combination, under laboratory and field conditions, against *R. dominica*, *T. castaneum*, *S. granarius*, and *T. granarium* adults on stored wheat. In the case of laboratory tests, mortality and progeny production was evaluated. In field studies, mortalities of the exposed individuals were estimated. Finally, the residual effect of the combined application of *B. bassiana* and spinetoram was conducted under laboratory trials.

## Materials and methods

### Rearing of test insects

Unsexed *T. castaneum*, *R. dominica*, *S. granarius*, and *T. granarium* adults were obtained from the Microbial Control Laboratory, Department of Entomology, University of Agriculture, Faisalabad, where they have been maintained for > 10 years unexposed to any insecticidal treatment. The individuals of *R. dominica*, *T. castaneum*, and *S. granarius* were < 2 weeks old while the individuals of *T. granarium* were < 24 h. *Rhyzopertha dominica*, *S. granarius*, and *T. granarium* were reared on whole wheat while *T. castaneum* was reared on wheat flour and 5% brewer’s yeast. All insect species were cultured at 30 °C, 65% relative humidity (RH), and 24 h darkness.

### Test grain

Untreated, clean, and free of insect and pathogen infestation soft wheat, *Triticum aestivum* L. (var. Noor 2013), was used in the trials. The wheat moisture content was 11.6% which was determined by using Dickey-John moisture meter (Dickey-John Multigrain CAC II, Dickey-John Co., Auburn, IL, USA).

### Culture of entomopathogenic fungi

*Beauveria bassiana* was inoculated in dishes (100 mm) containing Sabouraud Dextrose Agar (SDA) (Sigma-Aldrich Chemie GmbH, Taufkirchen, Germany). The dishes were wrapped with parafilm and placed into incubator (MIR-254, Panasonic, Japan) set at 25 °C under 14:10 h (light:dark) photoperiod for 10 days (Usman et al. 2020). The formed layers of conidia were harvested with a sterile scalpel on the surface of the SDA and transferred in a 50-ml falcon tube filled with 30 ml of sterile solution 0.05% Tween 80 (Merck, Kenilworth, NJ, USA). The conidia suspension was vortexed (Classic Vortex Mixer, Velp Scientifica Srl, Usmate Velate, Italy) with the addition of eight sterile glass beads for 5 min. The desired concentrations 1 × 10^7^ conidia/ml (for trials in the laboratory) and 1 × 10^9^ conidia/ml (for trials in the field) were determined with a Neubauer-improved hemocytometer (Marienfeld, Lauda-Königshofen, Germany) under the microscope. Conidia germination was determined by inoculating 0.1 ml (1 × 10^6^ conidia/ml) of solution on two dishes (60 mm) containing SDA + yeast. The dishes were wrapped with parafilm and transferred to incubator set at 25 °C and 14:10 h (light:dark) photoperiod for a period of 16 h. A sterile cover slip was put on dishes after incubation. Two countings were conducted by examining 200 conidia in each dish. Conidia were considered germinated when the germ tube > conidia (Inglis et al. 2012; Usman et al. 2020; Gulzar et al. 2021) at 400 × magnification under a Euromex BB.1152-PLi microscope (Euromex Microscopen bv, Arnhem, The Netherlands). The conidia viability was > 94% prior the initiation of the trials.

### Spinetoram

The insecticidal formulation Radiant^®^ 120 SC (Arysta LifeScience Pvt. Limited, Karachi, Pakistan), containing 11.7% spinetoram active ingredient (a.i.) that is a mixture of the compounds spinetoram-J and spinetoram-L, was used in the trials.

### Mortality and progeny in the laboratory

Experiments consisted of five treatments and control: *B. bassiana* alone at 1 × 10^7^ conidia/kg wheat (Bb), spinetoram alone at 0.05 ppm (Spine1) and 0.1 ppm (Spine2), *B. bassiana* plus spinetoram (Bb + spine1 and Bb + spine2), and control. Lots of 1 kg wheat were placed as thin layers on different trays to be treated, to maximize insecticide distribution in the entire mass of the kernels (Chanbang et al. 2008; Athanassiou et al. 2011). The applied doses of spinetoram were based on Vassilakos et al. (2012). An additional lot of 1 kg wheat was treated with 1 ml of 0.05% Tween 80 serving as control. Both *B. bassiana* and spinetoram were applied as liquids. Wheat was treated with 1 ml of the conidia suspension of *B. bassiana* or with 1 ml of an aqueous solution that contained the appropriate volume of spinetoram. An airbrush (Master Multipurpose Airbrush, USA) was used to spray the stored wheat quantities. The conidia suspensions, spinetoram, and Tween 80 were sprayed with different airbrushes. The sprayed wheat lots were transferred to different 3-l glass jars and shaken for 10 min manually to further attain the uniform distribution of the conidia, spinetoram, or Tween 80, in the whole wheat quantity (Chanbang et al. 2008; Athanassiou et al. 2011). Concerning combinations, spinetoram was applied first followed by the conidia suspension. The treated wheat lots were placed inside 3-l glass jars separately and shaken as described above. From each jar, three quantities of 100 g wheat were obtained. These quantities were weighted separately with an ELB 300 Shimadzu (Kyoto, Japan) compact balance on different layers. Then, the samples were placed in plastic vials (11-cm height, 6.5-cm diameter). To avoid the escape of insects, the internal upper edge of each vial had been coated with polytetrafluoroethylene (60 wt% dispersion in water) (Sigma-Aldrich Chemie GmbH, Taufkirchen, Germany). Fifty adults of *R. dominica* were released in each vial. Aeration of the vials was maintained because the lids of the vials had a central circular opening (1.5-cm diameter) covered by muslin cloth. The vials were kept in incubators set at 20 °C and 65% RH. Mortality was assessed 7 and 14 days post-exposure under a Leica Wild M3B stereomicroscope (Heerbrugg, Switzerland) at 40 × magnification. Per exposure interval, separate series of vials were prepared. At the 14-day count, all insect individuals were removed from the wheat. Wheat was put back in the vials and vials were returned back to the incubators at the same abiotic conditions to record the progeny production after 62 days (Wakil et al. 2021b). The entire procedure was replicated three times by preparing new wheat lots and vials. The same procedure was followed for 25 and 30 °C at 65% RH. Similarly, the aforementioned process was conducted for *S. granarius*, *T. castaneum*, and *T. granarium*. Offspring emergence was recorded after 62, 45, and 46 days for *T. castaneum* (Wakil et al. 2021b), *S. granarius* (Athanassiou et al. 2007), and *T. granarium* (Kavallieratos et al. 2017), respectively. Progeny production of *R. dominica* and *S. granarius* were adults, since their immatures are developed within kernels (Aitken 1975), while progeny of *T. confusum* and *T. granarium* were adults and immatures.

### Persistence in the laboratory

For a period of 180 days, the impact of the treatments Bb, spine1, spine2, Bb + spine1, Bb + spine2, and control was estimated against *R. dominica*, *S. granarius*, *T. castaneum*, and *T. granarium*, after 7 and 14 days of exposure. For this purpose, mortality of the exposed adults and their progeny emergence were realized every 30 days 30 °C and 65% RH as aforementioned. For the whole experimental period, the treated lots and controls were maintained in glass jars in a chamber set at 30 °C and 65% RH.

### Persistence in the field

Treatments were conducted by using *B. bassiana* at 1 × 10^9^ conidia/kg wheat and spinetoram at 0.75 and 1.25 ppm. The selection of the doses of spinetoram was based on preliminary experimentation. Combinations were the same as in the previous laboratory trials. Thirty 1-kg wheat lots were each spread on different polyethylene sheets and sprayed as described in the laboratory trials (6 treatments (including control) × 4 species × 30 kg wheat). Then, the sprayed wheat portions were mixed with a clean shovel (Wakil and Schmitt 2015) to make lots of 30 kg per treatment and control. New shovel was used to mix the grains per treatment or control. Subsequently, each lot was portioned in three quantities of 10 kg wheat each and placed in polypropylene bags. The bags were sealed, labeled, and transferred in a house-type storage facility in Punjab, Pakistan. This practice commonly followed for grains that will be stored in this area (Wakil and Schmitt 2015). Thirty days post-storage, each group of the six treatments (6 treatments × 3 bags × 10 kg wheat) was separately infested with 500 adults of *R. dominica*, *S. granarius*, *T. castaneum*, or *T. granarium*. After 14 days of treatment, from five different points (one from the center and four from the corners) of each bag, 200 g of wheat was taken with a grain sampler to make a sample of 1 kg (Wakil and Schmitt 2015). Per treatment, different grain samplers were used. Sampling was carried on every 30 days for a period of 180 days. The mortality data were estimated by counting living and dead adult insect individuals. The entire experiment was replicated three times by assembling new wheat portions, new bags that were stored in new house type stored rooms in Punjab. Average temperature and RH ranged between 26–35 °C and 55–67% respectively during the experimental period (HTC-2 electronic temperature and humidity meter, Shenzen Wanptek Electronic Co, Ltd., Shenzen, China).

### Data analysis

#### Mortality and progeny in the laboratory

Mortalities in controls were adjusted by using Abbott’s formula (Abbott 1925). The values were log (*x* + 1) transformed prior to analysis to normalize the variance (Zar 2014; Scheff and Arthur 2018). For each insect species, mortalities were submitted to a three-way analysis of variance (ANOVA) with treatment, temperature, and exposure interval as main effects. Mortality was the response variable. The associated interactions of the main effects were included in the analysis. For progeny production, data were submitted to a two-way ANOVA with treatment and temperature as main effects. Number of offspring was the response variable. The associated interaction of the main effects was incorporated in the analysis. Progeny in the control vials was incorporated into the analysis. The software Minitab 17 software (Minitab 2010) was used to analyze the data. Tukey–Kramer (HSD) test was used to compare means of mortalities and progeny at 5% significance level (Sokal and Rohlf 1995).

#### Persistence in the laboratory

Mortalities in controls were adjusted by using Abbott’s formula (Abbott 1925). The values were log (*x* + 1) transformed prior to analysis to normalize the variance (Zar 2014; Scheff and Arthur 2018). For each species and exposure, mortalities were submitted to a two-way ANOVA with treatment and storage period as the main effects. Mortality was the response variable. The associated interaction of the main effects was considered in the analysis. For progeny emergence, data were submitted to a two-way ANOVA with storage period and treatment as the main effects. Number of progeny was the response variable. The associated interaction of the main effects was incorporated into the analysis. Offspring in control vials was considered in the analysis. The software Minitab 17 software (Minitab 2010) was used to analyze the data. Tukey–Kramer (HSD) test was used to compare means of mortalities and progeny at 5% significance level (Sokal and Rohlf 1995).

#### Persistence in the field

Mortalities in controls were adjusted by using Abbott’s formula (Abbott 1925). The values were log (*x* + 1) transformed prior to analysis to normalize the variance (Zar 2014; Scheff and Arthur 2018). For each species, mortalities were submitted to a two-way ANOVA with treatment and storage period as main effects. Mortality was the response variable. The associated interaction of the main effects was added in the analysis. The software Minitab 17 software (Minitab 2010) was used to analyze the data. Tukey–Kramer (HSD) test was used to compare means of mortalities and progeny at 5% significance level (Sokal and Rohlf 1995).

## Results

### Mortality and progeny in the laboratory

Regarding *R. dominica*, all main effects and the interactions treatment × temperature, treatment × exposure interval, and temperature × exposure interval were significant (Table 1). The single application of the higher dose of spinetoram to wheat killed significantly higher adults than *B. bassiana* alone at all temperatures and exposure intervals (Table 2). Significantly higher mortalities were observed at 7 (78.8%) and 14 (89.8%) days post-exposure on wheat treated with the combined application of *B. bassiana* plus the higher dose of spinetoram at 30 °C compared to 25 and 20 °C (Table 2). Both combined applications produced significantly higher mortalities than single treatments at any temperature and exposure.

**Table 1:** ANOVA parameters of main effects and associated interactions for mortalities of *R. dominica*, *S. granarius*, *T. castaneum*, and *T. granarium* adults in laboratory trials (total *df* = 269 for all species)

Species		*R. dominica*		*S. granarius*		*T. castaneum*		*T. granarium*	
Source	*df*	*F*	*P*	*F*	*P*	*F*	*P*	*F*	*P*
Treatment	4	416.8	< 0.01	431.2	< 0.01	451.0	< 0.01	384.5	< 0.01
Temperature	2	208.5	< 0.01	185.1	< 0.01	201.4	< 0.01	174.6	< 0.01
Exposure interval	1	393.5	< 0.01	330.9	< 0.01	399.6	< 0.01	299.6	< 0.01
Treatment × temperature	8	7.0	< 0.01	1.7	0.09	2.4	0.01	3.5	< 0.01
Treatment × exposure interval	4	25.3	< 0.01	20.0	< 0.01	27.8	< 0.01	23.8	< 0.01
Temperature × exposure interval	2	4.4	0.01	0.1	0.87	1.1	0.35	0.4	0.65
Treatment × temperature × exposure interval	8	1.2	0.27	0.6	0.74	1.3	0.24	0.3	0.97

**Table 2:** Mean mortality (% ± SE) of *R. dominica*, *S. granarius*, *T. castaneum*, and *T. granarium* adults exposed for 7 and 14 days on wheat treated with one dose of *B. bassiana* (Bb: 1 × 10^7^ conidia/kg wheat), two doses of spinetoram (spine1: 0.05 ppm; spine2: 0.1 ppm), and their combinations (Bb + spine1; Bb + spine2) under three temperatures for 7 and 14 days in laboratory trials

Species	Treatment	Exposure interval: 7 days					Exposure interval: 14 days				
		Temperature					Temperature				
		20 °C	25 °C	30 °C	*F*	*P*	20 °C	25 °C	30 °C	*F*	*P*
*R. dominica*	Bb	16.5 ± 3.6 Bd	24.6 ± 2.8 Ad	30.7 ± 3.5 Ad	21.0	< 0.01	29.2 ± 2.8 Bc	45.6 ± 3.7 Ac	52.0 ± 2.3 Ac	26.4	< 0.01
	Spine1	25.4 ± 2.7 Bc	32.1 ± 2.0 Ac	38.2 ± 2.3 Ac	10.5	< 0.01	34.3 ± 2.3 Cc	51.8 ± 3.1 Bc	65.7 ± 3.3 Ab	65.4	< 0.01
	Spine2	34.5 ± 3.7 Bb	40.3 ± 2.6 Bb	49.3 ± 2.0 Ab	13.3	< 0.01	42.7 ± 2.4 Bb	64.5 ± 2.8 Ab	72.2 ± 2.2 Ab	56.7	< 0.01
	Bb + spine1	56.2 ± 2.8 Ca	65.2 ± 3.3 Ba	72.7 ± 3.9 Aa	17.7	< 0.01	63.7 ± 3.5 Ba	76.5 ± 2.0 Aa	81.2 ± 3.7 Aa	20.0	< 0.01
	Bb + spine2	63.3 ± 3.9 Ba	70.2 ± 2.3 Ba	78.8 ± 4.2 Aa	13.0	< 0.01	72.2 ± 3.7 Ca	82.9 ± 4.2 Ba	89.8 ± 3.3 Aa	29.5	< 0.01
	*F*	103.0	65.8	107.0			58.7	56.6	51.7		
	*P*	< 0.01	< 0.01	< 0.01			< 0.01	< 0.01	< 0.01		
*S. granarius*	Bb	15.6 ± 1.0 Bd	21.3 ± 2.4 Ad	25.3 ± 2.1 Ad	16.9	< 0.01	26.9 ± 2.3 Bc	37.0 ± 2.1 Ad	41.5 ± 3.2 Ad	13.5	< 0.01
	Spine1	20.4 ± 2.3 Bc	29.5 ± 1.8 Ac	33.1 ± 3.2 Ac	13.6	< 0.01	31.7 ± 2.2 Bc	45.3 ± 3.7 Ac	50.6 ± 3.6 Ac	20.3	< 0.01
	Spine2	31.7 ± 3.1 Bb	39.6 ± 3.7 Ab	45.2 ± 3.0 Ab	13.6	< 0.01	40.5 ± 2.9 Bb	58.4 ± 3.4 Ab	64.8 ± 2.9 Ab	48.1	< 0.01
	Bb + spine1	47.5 ± 1.7 Ba	58.6 ± 3.7 Aa	65.5 ± 3.5 Aa	16.2	< 0.01	56.1 ± 3.3 Ca	71.3 ± 2.2 Ba	77.9 ± 1.3 Aa	47.1	< 0.01
	Bb + spine2	55.2 ± 2.8 Ba	69.3 ± 2.5 Aa	76.7 ± 2.9 Aa	29.5	< 0.01	62.8 ± 3.2 Ca	78.5 ± 2.3 Ba	85.8 ± 3.2 Aa	47.4	< 0.01
	*F*	112.0	72.2	75.1			47.6	64.0	77.5		
	*P*	< 0.01	< 0.01	< 0.01			< 0.01	< 0.01	< 0.01		
*T. castaneum*	Bb	10.2 ± 1.3 Bd	18.1 ± 1.3 Ad	21.6 ± 2.8 Ad	22.7	< 0.01	24.8 ± 2.7 Bc	32.3 ± 3.3 Ae	39.4 ± 2.8 Ae	15.2	< 0.01
	Spine1	15.8 ± 2.8 Bc	26.0 ± 31.9 Ac	30.2 ± 2.9 Ac	21.1	< 0.01	27.8 ± 2.0 Bc	40.6 ± 3.0 Ad	46.2 ± 3.1 Ad	18.4	< 0.01
	Spine2	30.7 ± 3.2 Bb	37.3 ± 2.7 Ab	43.6 ± 2.1 Ab	12.9	< 0.01	40.2 ± 2.9 Bb	53.6 ± 1.6 Ac	57.1 ± 2.7 Ac	24.8	< 0.01
	Bb + spine1	41.3 ± 2.8 Ca	52.7 ± 2.3 Ba	61.8 ± 3.7 Aa	21.1	< 0.01	49.2 ± 3.3 Cab	64.2 ± 2.2 Bb	73.5 ± 2.9 Ab	40.9	< 0.01
	Bb + spine2	49.4 ± 2.2 Ba	61.3 ± 3.7 Aa	68.9 ± 3.1 Aa	22.5	< 0.01	56.3 ± 2.6 Ba	77.3 ± 2.8 Aa	81.6 ± 3.4 Aa	48.0	< 0.01
	*F*	118.0	86.0	76.7			43.4	61.6	81.4		
	*P*	< 0.01	< 0.01	< 0.01			< 0.01	< 0.01	< 0.01		
*T. granarium*	Bb	8.3 ± 1.3 Bd	14.4 ± 1.0 Ad	18.6 ± 2.2 Ad	13.6	< 0.01	17.9 ± 2.9 Cd	28.1 ± 2.2 Bd	36.0 ± 3.1 Ac	29.5	< 0.01
	Spine1	13.5 ± 2.5 Bc	20.1 ± 2.9 Ac	25.2 ± 2.3 Ac	14.7	< 0.01	24.5 ± 3.1 Bc	34.0 ± 3.6 Ac	40.5 ± 2.3 Ac	18.5	< 0.01
	Spine2	27.7 ± 2.7 Cb	33.4 ± 2.7 Bb	40.8 ± 3.3 Ab	17.6	< 0.01	35.4 ± 2.1 Cb	48.8 ± 2.8 Bb	55.6 ± 3.9 Ab	41.7	< 0.01
	Bb + spine1	36.9 ± 2.5 Bab	45.4 ± 3.8 Aa	52.4 ± 2.3 Aab	13.4	< 0.01	43.3 ± 2.1 Bab	54.8 ± 2.1 Ab	61.1 ± 2.0 Ab	20.9	< 0.01
	Bb + spine2	43.2 ± 2.7 Ca	56.4 ± 2.1 Ba	64.9 ± 2.2 Aba	31.0	< 0.01	50.3 ± 3.0 Ba	67.4 ± 2.4 Aa	75.5 ± 3.0 Aa	31.6	< 0.01
	*F*	69.7	93.3	71.7			51.8	59.2	59.9		
	*P*	< 0.01	< 0.01	< 0.01			< 0.01	< 0.01	< 0 .01		

For each species and exposure interval, within each treatment, means followed by the same uppercase letter are not significantly different; *df* = 2, 26, Tukey–Kramer (HSD) test at *P* = 0.05. For each species and exposure interval, within each temperature, means followed by the same lowercase letter are not significantly different; *df* = 4, 44, Tukey–Kramer (HSD) test at *P* = 0.05

For *S. granarius*, all main effects and the interaction treatment × exposure interval were significant (Table 1). At 30 °C, the grains treated with single application of each agent caused < 46% mortality 7 days post-application (Table 2). The maximum mortalities, i.e., 76.7 and 65.5%, were observed at the same temperature and exposure interval on wheat treated with the combined application of *B. bassiana* plus the higher and the lower dose of spinetoram, respectively. Further, an increasing trend of the overall mortality was observed after 14 days of exposure reaching the highest value of 85.8% when *B. bassiana* was combined with spinetoram at the higher dose. Both tested combinations caused significantly higher mortalities than the applications of the fungus and spinetoram alone at all temperatures and exposure intervals.

Concerning *T. castaneum*, all main effects and the associated interactions treatment × temperature and treatment × exposure interval were significant (Table 1). The overall mortality ranged between 10.2 and 68.9% 7 days post-exposure (Table 2). Although mortality was further increased after 14 days of exposure, it remained < 78% on wheat treated with the *B. bassiana* plus the higher dose of spinetoram at 25 °C. However, at 30 °C, mortality at the same combination reached 81.6%. At 25 and 30 °C, mortalities were significantly higher on wheat treated with *B. bassiana* plus the higher dose of spinetoram than mortalities of all other treatments.

In the case of *T. granarium*, all main effects and the associated interactions treatment × temperature and treatment × exposure interval were significant (Table 1). At 30 °C, only the combination of *B. bassiana* plus spinetoram at the higher dose provided mortality > 64% 7 days post-application (Table 2). After 14 days of treatment application, the overall mortality was further increased but it did not exceed 67.4% at 25 °C. When temperature increased to 30 °C, 75.5% of the exposed individuals died on wheat treated with *B. bassiana* plus the higher dose of spinetoram, a value that was significantly higher than mortality values of all other treatments.

Regarding progeny production of all tested species, all main effects and the associate interaction were significant (Table 3). Offspring emergence was significanty lower in treatments than in controls at all temperatures (Table 4). Both combinations resulted to significantly lower progeny populations than single treatments at any temperature. Significantly less *S. granarius* and *T. castaneum* progeny were recorded in the combination of *B. bassiana* plus the higher dose of spinetoram compared to *B. bassiana* plus the lower dose of spinetoram at all temperatures. The increase of temperature decreased the appearance of offspring significantly in the majority of single and combined treatments.

**Table 3:** ANOVA parameters of main effects and associated interactions for progeny production of *R. dominica*, *S. granarius*, *T. castaneum*, and *T. granarium* individuals in laboratory trials (total *df* = 161 for all species)

Species		*R. dominica*		*S. granarius*		*T. castaneum*		*T. granarium*	
Source	*df*	*F*	*P*	*F*	*P*	*F*	*P*	*F*	*P*
Treatment	5	359.7	< 0.01	367.6	< 0.01	417.5	< 0.01	527.3	< 0.01
Temperature	2	51.5	< 0.01	63.3	< 0.01	172.9	< 0.01	204.8	< 0.01
Treatment × temperature	10	9.7	< 0.01	8.7	< 0.01	14.9	< 0.01	14.8	< 0.01

**Table 4:** Mean number (± SE) of *R. dominica*, *S. granarius*, *T. castaneum*, and *T. granarium* individuals per vial following a 14-day exposure interval of parents on wheat treated with one dose of *B. bassiana* (Bb: 1 × 10^7^ conidia/kg wheat), two doses of spinetoram (spine1: 0.05 ppm; spine2: 0.1 ppm), and their combinations (Bb + spine1; Bb + spine2) and control under three temperatures in laboratory trials

Species	Treatment	Temperature				
		20 °C	25 °C	30 °C	*F*	*P*
*R. dominica*	Bb	92.7 ± 1.9 Aa	77.8 ± 1.9 Bb	60.1 ± 1.8 Cb	70.4	< 0.01
	Spine1	75.2 ± 2.0 Ab	63.4 ± 2.9 Bc	51.4 ± 3.4 Cbc	19.8	< 0.01
	Spine2	61.1 ± 1.9 Ac	52.2 ± 3.8 Bd	42.2 ± 1.6 Cc	18.2	< 0.01
	Bb + spine1	45.0 ± 3.3 Ad	39.4 ± 1.2 Ae	31.3 ± 2.5 Bd	24.1	< 0.01
	Bb + spine2	33.2 ± 2.2 Ae	28.8 ± 1.4 Af	26.3 ± 1.3 Ad	1.0	< 0.01
	Control	110.8 ± 2.6 Ba	118.1 ± 3.7 Ba	132.5 ± 2.6 Aa	12.8	< 0.01
	*F*	94.9	163.0	132.0		
	*P*	< 0.01	< 0.01	< 0.01		
*S. granarius*	Bb	99.2 ± 2.5 Aa	81.3 ± 2.9 Bb	69.3 ± 3.4 Cb	23.1	< 0.01
	Spine1	81.2 ± 2.6 Ab	65.1 ± 3.4 Bc	54.7 ± 2.7 Cc	19.0	< 0.01
	Spine2	63.2 ± 3.9 Ac	53.8 ± 1.5 ABd	48.2 ± 1.3 Bc	8.6	< 0.01
	Bb + spine1	52.7 ± 1.4 Ad	45.2 ± 1.3 Be	37.1 ± 1.8 Cd	23.2	< 0.01
	Bb + spine2	39.6 ± 1.1 Ae	37.8 ± 2.1 Af	26.6 ± 1.7 Be	17.8	< 0.01
	Control	115.1 ± 2.6 Ca	121.4 ± 3.0 Ba	133.1 ± 1.4 Aa	18.3	< 0.01
	*F*	109.0	117.0	150.0		
	*P*	< 0.01	< 0.01	< 0.01		
*T. castaneum*	Bb	71.5 ± 3.0 Ab	52.4 ± 3.1 Bb	43.5 ± 1.5 Cb	29.1	< 0.01
	Spine1	62.3 ± 3.9 Ab	43.0 ± 2.2 Bbc	34.0 ± 0.9 Cc	36.7	< 0.01
	Spine2	49.3 ± 2.0 Ac	37.1 ± 1.6 Bc	26.0 ± 1.1 Cd	51.5	< 0.01
	Bb + spine1	38.7 ± 1.7 Ad	25.4 ± 1.2 Bd	19.1 ± 1.3 Ce	47.0	< 0.01
	Bb + spine2	30.2 ± 1.4 Ae	20.4 ± 1.2 Be	13.3 ± 1.0 Cf	46.9	< 0.01
	Control	89.2 ± 2.3 Ba	94.5 ± 0.9 Ba	108.9 ± 3.8 Aa	15.4	< 0.01
	*F*	76.1	132.0	235.0		
	*P*	< 0.01	< 0.01	< 0.01		
*T. granarium*	Bb	191.0 ± 3.1 Ab	162.4 ± 3.5 Bb	139.5 ± 3.8 Cb	49.0	< 0.01
	Spine1	165.4 ± 3.4 Ac	138.0 ± 5.0 Bc	121.8 ± 2.7 Cc	30.5	< 0.01
	Spine2	147.4 ± 4.3 Ad	116.4 ± 2.4 Bd	103.6 ± 3.6 Cd	38.8	< 0.01
	Bb + spine1	129.1 ± 3.9 Ae	106.0 ± 3.7 Bde	81.9 ± 2.3 Ce	61.3	< 0.01
	Bb + spine2	110.3 ± 2.8 Af	96.8 ± 2.1 Be	69.7 ± 1.4 Cf	60.6	< 0.01
	Control	229.6 ± 2.7 Ba	236.3 ± 1.7 ABa	244.5 ± 3.7 Aa	4.9	< 0.01
	*F*	108.0	157.0	292.0		
	*P*	< 0.01	< 0.01	< 0.01		

For each species, within each treatment, means followed by the same uppercase letter are not significantly different; *df* = 2, 26, Tukey–Kramer (HSD) test at *P* = 0.05. For each species, within each temperature, means followed by the same lowercase letter are not significantly different; *df* = 5, 53, Tukey–Kramer (HSD) test at *P* = 0.05

### Mortality and progeny in the laboratory persistence trials

For *R. dominica*, all main effects were significant after 7 and 14 days of exposure (Table 5). For the entire storage period, the combinations of *B. bassiana* plus spinetoram produced significantly higher mortalities than single treatments (Table 6) 7 days post-exposure. The same trend was noticed for 60 days of storage period and from the 120- to the 180-day trial after 14 days of exposure (Table 7). The 30-day of storage mortality reached 82.8% on wheat treated with *B. bassiana* plus the higher dose of spinetoram 14 days post-exposure. Although the mortalities gradually decreased in all treatments during the storage period, > 62% of the exposed individuals died after 120 days of storage due to the combination of *B. bassiana* plus the higher dose of spinetoram 14 days post-exposure. Even after the 180-day trial, mortality was 50.7% at the same combination and post-exposure interval.

**Table 5:** ANOVA parameters of main effects and associated interactions for mortalities of *R. dominica*, *S. granarius*, *T. castaneum*, and *T. granarium* adults in each exposure interval in laboratory persistence trials (total *df* = 314 for all species)

Species	Exposure interval		7 days		14 days	
	Source	*df*	*F*	*P*	*F*	*P*
*R. dominica*	Treatment	4	8.3	< 0.01	674.9	< 0.01
	Storage period	6	4.2	< 0.01	256.7	< 0.01
	Treatment × storage period	24	0.1	1.000	1.3	0.17
*S. granarius*	Treatment	4	957.0	< 0.01	187.9	< 0.01
	Storage period	6	228.9	< 0.01	520.3	< 0.01
	Treatment × storage period	24	2.3	< 0.01	1.5	0.11
*T. castaneum*	Treatment	4	800.7	< 0.01	800.2	< 0.01
	Storage period	6	190.0	< 0.01	264.5	< 0.01
	Treatment × storage period	24	2.3	< 0.01	3.0	< 0.01
*T. granarium*	Treatment	4	866.5	< 0.01	7.7	< 0.01
	Storage period	6	245.0	< 0.01	0.3	0.94
	Treatment × storage period	24	6.5	< 0.01	1.5	0.11

**Table 6:** Mean mortality (% ± SE) of *R. dominica*, *S. granarius*, *T. castaneum*, and *T. granarium* adults exposed for 7 days on wheat treated with one dose of *B. bassiana* (Bb: 1 × 10^7^ conidia/kg wheat), two doses of spinetoram (spine1: 0.05 ppm; spine2: 0.1 ppm), and their combinations (Bb + spine1; Bb + spine2) in seven laboratory trials carried out from 0 to 180 days after treatment

Species	Treatment	Trials at a given number of days after treatment								
		0	30	60	90	120	150	180	*F*	*P*
*R. dominica*	Bb	33.6 ± 2.6 Ae	28.2 ± 2.9 ABd	24.2 ± 2.3 ABd	20.7 ± 2.1 BCd	16.2 ± 1.1 CDd	12.7 ± 2.5 DEd	9.9 ± 2.7 Ec	28.4	< 0.01
	Spine1	41.8 ± 1.7 Ad	37.3 ± 3.5 Ac	31.5 ± 3.1 ABc	27.8 ± 2.4 BCc	22.4 ± 2.8 CDc	18.4 ± 3.7 Dc	13.6 ± 2.0 Ec	33.8	< 0.01
	Spine2	58.9 ± 2.7 Ac	52.3 ± 3.2 ABb	47.0 ± 2.0 ABCb	41.9 ± 3.2 BCb	37.4 ± 2.9 CDb	32.0 ± 3.6 DEb	27.8 ± 2.0 Eb	19.8	< 0.01
	Bb + spine1	71.7 ± 1.2 Ab	64.8 ± 3.7 ABa	60.2 ± 3.9 BCa	55.3 ± 2.2 Ca	51.6 ± 2.1 CDa	45.4 ± 2.2 DEa	41.4 ± 2.6 Ea	27.7	< 0.01
	Bb + spine2	84.1 ± 3.2 Aa	72.5 ± 2.3 Ba	67.2 ± 1.8 BCa	61.3 ± 1.1 CDa	57.1 ± 3.0 DEa	50.9 ± 3.1 EFa	46.8 ± 3.3 Fa	37.9	< 0.01
	*F*	104.0	76.1	69.9	70.1	62.6	105.0	61.9		
	*P*	< 0.01	< 0.01	< 0.01	< 0.01	< 0.01	< 0.01	< 0.01		
*S. granarius*	Bb	27.4 ± 2.7 Ac	23.8 ± 1.4 Ad	20.6 ± 3.6 ABc	15.1 ± 1.3 BCd	12.3 ± 1.9 Cd	9.5 ± 2.9 CDc	7.5 ± 1.1 Dc	23.0	< 0.01
	Spine1	32.8 ± 3.4 Ac	28.8 ± 2.9 Ac	24.9 ± 2.0 ABc	20.1 ± 1.6 BCc	15.9 ± 1.6 Cc	11.1 ± 3.2 Dc	9.3 ± 1.4 Dc	36.1	< 0.01
	Spine2	49.7 ± 2.9 Ab	45.8 ± 3.2 ABb	41.7 ± 2.7 ABb	35.0 ± 2.6 BCb	31.2 ± 3.2 CDb	25.7 ± 2.9 DEb	21.6 ± 2.1 Eb	24.2	< 0.01
	Bb + spine1	67.4 ± 3.5 Aa	62.3 ± 2.7 Aa	57.3 ± 2.5 ABa	50.5 ± 3.2 BCa	43.5 ± 2.1 CDa	38.1 ± 3.0 DEa	33.6 ± 2.8 Ea	36.7	< 0.01
	Bb + spine2	75.6 ± 3.0 Aa	68.7 ± 3.1 ABa	62.3 ± 2.5 BCa	57.7 ± 4.0 CDa	51.7 ± 3.1 DEa	45.3 ± 2.9 EFa	40.7 ± 1.2 Fa	36.0	< 0.01
	*F*	72.2	103.0	107.0	63.7	103.0	63.5	65.8		
	*P*	< 0.01	< 0.01	< 0.01	< 0.01	< 0.01	< 0.01	< 0.01		
*T. castaneum*	Bb	22.0 ± 2.9 Ad	19.8 ± 2.7 ABc	16.3 ± 2.7 ABc	12.3 ± 2.5 BCd	9.1 ± 1.3 CDc	6.8 ± 0.7 DEc	4.3 ± 0.4 Ed	27.6	< 0.01
	Spine1	28.3 ± 3.1 Ac	24.3 ± 3.8 Ac	18.4 ± 3.1 Bc	15.7 ± 2.1 BCc	12.1 ± 2.9 CDc	10.0 ± 1.4 Dc	7.0 ± 1.3 Dc	36.2	< 0.01
	Spine2	44.6 ± 3.4 Ab	38.3 ± 2.4 ABb	32.4 ± 4.1 BCb	27.3 ± 2.0 CDb	23.2 ± 2.8 Deb	18.2 ± 2.6 Eb	15.9 ± 1.2 Eb	23.5	< 0.01
	Bb + spine1	61.1 ± 3.6 Aa	55.7 ± 3.3 ABa	50.1 ± 3.7 BCa	44.2 ± 2.5 CDa	38.0 ± 2.1 DEa	34.7 ± 2.5 Ea	29.3 ± 2.6 Ea	25.1	< 0.01
	Bb + spine2	65.4 ± 2.7 Aa	60.2 ± 2.7 ABa	56.2 ± 2.7 BCa	50.3 ± 2.9 CDa	43.7 ± 2.5 DEa	39.2 ± 3.3 Ea	35.2 ± 2.4 Ea	27.5	< 0.01
	*F*	94.3	83.3	154.0	110.0	51.3	51.5	70.2		
	*P*	< 0.01	< 0.01	< 0.01	< 0.01	< 0.01	< 0.01	< 0 .01		
*T. granarium*	Bb	17.2 ± 1.6 Ad	14.1 ± 1.8 ABe	12.2 ± 1.4 ABc	8.2 ± 1.1 BCd	6.4 ± 1.1 CDd	4.7 ± 0.7 CDb	3.3 ± 0.5 Dc	15.7	< 0.01
	Spine1	21.3 ± 2.1 Ad	18.2 ± 1.7 ABd	14.7 ± 2.0 ABCc	12.3 ± 1.3 BCc	9.6 ± 1.3 CDc	6.8 ± 1.29 DEb	5.1 ± 1.1 Ec	18.8	< 0.01
	Spine2	35.3 ± 2.8 Ac	31.6 ± 2.3 Ac	27.9 ± 2.4 ABb	22.3 ± 2.1 BCb	19.8 ± 2.5 CDb	15.9 ± 1.9 DEa	13.4 ± 1.9 Eb	24.8	< 0.01
	Bb + spine1	46.2 ± 3.2 Ab	40.9 ± 2.4 ABb	35.6 ± 3.0 ABCb	31.4 ± 2.2 BCab	27.6 ± 3.1 CDab	23.5 ± 1.2 DEa	19.7 ± 2.0 Eab	21.3	< 0.01
	Bb + spine2	57.9 ± 2.7 Aa	53.4 ± 2.5 Aa	46.5 ± 2.1 ABa	40.1 ± 2.3 BC1a	34.6 ± 2.3 CDa	28.1 ± 1.9 DEa	24.4 ± 1.0 Ea	32.3	< 0.01
	*F*	88.1	96.1	69.5	52.9	58.4	27.8	36.0		
	*P*	< 0.01	< 0.01	< 0.01	< 0.01	< 0.01	< 0.01	< 0.01		

For each species, within each treatment, means followed by the same uppercase letter are not significantly different; in all cases *df* = 6, 62 Tukey’s HSD test at *P* = 0.05. For each species, within each trial, means followed by the same lowercase letter are not significantly different; in all cases *df* = 4, 44 Tukey’s HSD test at *P* = 0.05

**Table 7:** Mean mortality (% ± SE) of adult of *R. dominica*, *S. granarius*, *T. castaneum*, and *T. granarium* exposed for 14 days on wheat treated with one dose of *B. bassiana* (Bb: 1 × 10^7^ conidia/kg wheat), two doses of spinetoram (spine1: 0.05 ppm; spine2: 0.1 ppm), and their combinations (Bb + spine1; Bb + spine2) in seven laboratory trials carried out from 0 to 180 days after treatment

Species	Treatment	Trials at a given number of days after treatment								
		0	30	60	90	120	150	180	*F*	*P*
*R. dominica*	Bb	44.5 ± 2.8 Ad	39.4 ± 3.7 Ad	34.9 ± 2.1 ABd	28.8 ± 2.7 BCd	24.8 ± 1.6 CDd	20.4 ± 1.6 Dec	17.8 ± 3.5 Ec	23.6	< 0.01
	Spine1	57.3 ± 2.8 Ac	51.5 ± 3.1 ABc	46.7 ± 2.6 ABc	40.3 ± 2.9 BCc	35.6 ± 3.4 Cc	26.6 ± 2.9 Dc	21.9 ± 2.8 Dc	32.6	< 0.01
	Spine2	68.8 ± 2.9 Ab	62.2 ± 2.1 ABb	56.7 ± 3.4 ABCb	51.7 ± 2.8 BCb	46.1 ± 2.8 CDb	37.9 ± 2.6 Deb	33.8 ± 2.0 Eb	29.0	< 0.01
	Bb + spine1	78.7 ± 3.0 Aa	73.2 ± 4.0 Aa	68.3 ± 2.7 ABa	62.5 ± 3.9 BCab	56.6 ± 2.8 CDa	50.3 ± 1.4 DEa	45.5 ± 2.3 Ea	37.5	< 0.01
	Bb + spine2	87.8 ± 3.7 Aa	82.8 ± 2.8 ABa	75.6 ± 2.6 BCa	68.2 ± 2.3 CDa	62.8 ± 3.5 DEa	56.5 ± 1.8 EFa	50.7 ± 3.0 Fa	44.3	< 0.01
	*F*	67.3	63.9	61.3	46.2	57.5	39.0	46.9		
	*P*	< 0.01	< 0.01	< 0.01	< 0.01	< 0.01	< 0.01	< 0.01		
*S. granarius*	Bb	39.3 ± 2.3 Ad	33.4 ± 2.8 ABd	29.9 ± 2.4 ABCd	25.9 ± 2.9 BCDd	22.6 ± 2.4 CDEd	19.4 ± 3.2 DEd	15.5 ± 3.9 Ec	15.4	< 0.01
	Spine1	50.1 ± 2.8 Ac	45.0 ± 3.5 Ac	40.2 ± 3.5 ABc	35.1 ± 3.3 BCc	30.6 ± 2.8 CDc	27.2 ± 2.6 Dc	24.8 ± 3.2 Db	21.2	< 0.01
	Spine2	62.3 ± 3.0 Ab	56.7 ± 3.3 ABb	51.2 ± 3.4 Bb	47.0 ± 3.0 BCb	41.7 ± 3.7 CDb	37.6 ± 2.1 Deb	31.3 ± 3.8 Eb	29.3	< 0.01
	Bb + spine1	74.7 ± 2.9 Aa	68.7 ± 3.5 ABa	60.9 ± 2.5 BCa	56.2 ± 2.1 CDab	50.2 ± 2.1 DEab	45.7 ± 3.8 EFab	40.1 ± 2.5 Fa	35.7	< 0.01
	Bb + spine2	83.7 ± 3.7 Aa	77.1 ± 3.9 ABa	69.4 ± 3.7 BCa	61.5 ± 3.2 CDa	56.2 ± 3.5 DEa	52.4 ± 3.1 EFa	47.7 ± 2.8 Fa	44.9	< 0.01
	*F*	60.3	51.4	59.3	49.5	31.5	51.9	53.1		
	*P*	< 0.01	< 0.01	< 0.01	< 0.01	< 0.01	< 0.01	< 0.01		
*T. castaneum*	Bb	33.8 ± 1.6 Ad	29.4 ± 2.6 Ac	25.3 ± 3.0 Ac	21.3 ± 1.3 ABc	19.7 ± 1.9 AB4c	16.7 ± 1.2 BCc	14.1 ± 1.2 Cc	11.4	< 0.01
	Spine1	40.5 ± 2.9 Ac	35.6 ± 2.6 ABc	31.3 ± 2.4 ABCc	25.7 ± 3.1 BCc	21.8 ± 1.2 CDc	18.6 ± 1.5 Dc	17.7 ± 1.4 Dc	15.7	< 0.01
	Spine2	56.2 ± 2.1 Ab	50.8 ± 3.0 ABb	45.4 ± 2.9 ABb	40.4 ± 2.8 BCb	35.4 ± 2.7 CDb	30.2 ± 1.4 Deb	25.6 ± 1.8 Eb	27.0	< 0.01
	Bb + spine1	70.1 ± 3.5 Aa	61.2 ± 2.3 ABa	56.9 ± 2.2 BCa	50.4 ± 3.5 Cab	46.6 ± 2.8 CDa	42.8 ± 1.4 DEab	35.7 ± 2.8 Eab	31.4	< 0.01
	Bb + spine2	75.5 ± 2.5 Aa	67.9 ± 2.6 ABa	63.5 ± 2.6 Ba	56.5 ± 3.0 BCa	50.3 ± 3.4 CDa	46.3 ± 2.7 DE4a	40.5 ± 1.6 Ea	34.2	< 0.01
	*F*	67.6	52.9	48.2	32.4	46.5	36.3	38.6		
	*P*	< 0.01	< 0.01	< 0.01	< 0.01	< 0.01	< 0.01	< 0.01		
*T. granarium*	Bb	28.4 ± 2.9 Ac	25.3 ± 2.6 Ac	21.3 ± 3.5 ABc	18.0 ± 1.1 ABc	14.2 ± 2.9 BCd	11.8 ± 2.7 CDc	9.3 ± 0.9 Dd	19.2	< 0.01
	Spine1	35.6 ± 3.2 Ac	30.8 ± 3.1 ABc	25.1 ± 2.0 ABCc	21.1 ± 3.1 BCc	18.2 ± 2.4 CDc	15.1 ± 1.7 Dec	12.8 ± 1.6 Ec	21.0	< 0.01
	Spine2	50.5 ± 2.0 Ab	45.3 ± 2.8 ABb	40.1 ± 3.4 ABCb	35.3 ± 2.5 BCDb	32.5 ± 2.1 CDb	27.4 ± 1.7 Deb	24.5 ± 1.5 Eb	19.6	< 0.01
	Bb + spine1	64.8 ± 3.2 Aa	55.9 ± 3.9 Aab	54.1 ± 2.09 ABab	42.2 ± 3.2 BCb	38.2 ± 2.7 Cb	34.8 ± 2.4 CDab	31.6 ± 1.8 Dab	23.2	< 0.01
	Bb + spine2	71.8 ± 2.8 Aa	67.0 ± 2.5 ABa	60.3 ± 3.2 BCa	55.7 ± 2.2 CDa	49.0 ± 1.1 CDa	44.8 ± 2.7 DEa	40.6 ± 1.3 Ea	29.6	< 0.01
	*F*	60.3	45.5	51.5	47.6	58.4	49.5	43.3		
	*P*	< 0.01	< 0.01	< 0.01	< 0.01	< 0.01	< 0.01	< 0.01		

For each species, within each treatment, means followed by the same uppercase letter are not significantly different; in all cases *df* = 6, 62 Tukey’s HSD test at *P* = 0.05. For each species, within each trial, means followed by the same lowercase letter are not significantly different; in all cases *df* = 4, 44 Tukey’s HSD test at *P* = 0.05

Regarding *S. granarius*, all main effects and their associated interaction were significant after 7 days of exposure, while all main effects were significant 14 days post-exposure (Table 5). Both combinations killed significantly more adults than single treatments during the whole storage period 7 days post-exposure (Table 6). Also, both combined treatments caused the death of significantly more adults for a period of 0, 30, 60, and 180 days after 14 days of exposure (Table 7). The highest mortality (83.7%) was observed in the combination of *B. bassiana* plus the higher dose of spinetoram at 0 day of trial 14 days post-exposure. Yet, at the 90-day trial, the mortality was 61.5% on wheat treated with the combination of *B. bassiana* plus the higher dose of spinetoram 14 days post-exposure. At the 180 days of storage period, the overall mortality ranged between 15.5 and 47.7%.

Concerning *T. castaneum*, all main effects and their associated interaction were significant after 7 and 14 days of exposure (Table 5). Significantly more adults died on wheat treated with the combination of *B. bassiana* plus the higher dose of spinetoram than the single treatments for the whole storage period 7 and 14 days post-exposure (Tables 6 and 7). The maximum mortality (75.5%) was observed at the first trial on wheat treated with *B. bassiana* plus the higher dose of spinetoram 14 days post-exposure (Table 7). As the storage time passed, the overall mortality was decreased for both exposure intervals. Until 60 days of storage, mortality was 63.5% at the combination that included the higher dose of spinetoram after 14 days of exposure. All single treatments provided mortalities < 50% between 60 and 180 days of trials. After 180 days of storage the highest mortality was < 41%.

As far as *T. granarium* is concerned, all main effects and their associated interaction were significant 7 days post-exposure while the main effect treatment was significant after 14 days of exposure (Table 5). This species exhibited the lowest mortality rates than all other tested species (Tables 6 and 7). The combination of *B. bassiana* plus the higher dose of spinetoram killed significantly more adults at all trials 14 days post-exposure (Table 7). The same treatment provided the highest adult mortality (71.8%) at the first trial. There was a considerable reduction in the overall mortality during the storage period 7 and 14 days post-exposure. The levels of mortality were low at the last trial for single treatments (i.e., range 9.3–24.5%) while they were moderate for the combined treatments (i.e., range 31.6–40.6%) 14 days post-exposure.

In the case of progeny, for *R. dominica*, all main effects were significant, while for *S. granarius*, *T. castaneum*, and *T. granarium*, all main effects and the associated interaction were significant (Table 8). The emergence of offspring individuals was significantly lower in all treatments compared to controls for all storage periods and species (Table 9). Significantly less progeny was noted on wheat treated with *B. bassiana* plus the higher dose of spinetoram than on wheat singly treated with *B. bassiana* or spinetoram at any storage period and insect species. The lowest overall progeny was detected at the 0 day of the storage period while it was increased with the increase of the storage time for all tested species. *Trogoderma granarium* exhibited the highest progeny production followed by *S. granarius* and *R. dominica*.

**Table 8:** ANOVA parameters of main effects and associated interactions for progeny production of *R. dominica, S. granarius, T. castaneum*, and *T. granarium* individuals in laboratory persistence trials (total *df* = 377 for all species)

Species	Source	*df*	*F*	*P*
*R. dominica*	Treatment	5	1303.0	< 0.01
	Storage period	6	364.7	< 0.01
	Treatment × storage period	30	1.4	0.14
*S. granarius*	Treatment	5	1788.6	< 0.01
	Storage period	6	709.4	< 0.01
	Treatment × storage period	30	2.7	< 0.01
*T. castaneum*	Treatment	5	1617.5	< 0.01
	Storage period	6	493.2	< 0.01
	Treatment × storage period	30	2.2	< 0.01
*T. granarium*	Treatment	5	1361.9	< 0.01
	Storage period	6	426.7	< 0.01
	Treatment × storage period	30	7.5	< 0.01

**Table 9:** Mean number (± SE) of *R. dominica*, *S. granarius*, *T. castaneum*, and *T. granarium* individuals per vial following a 14-day exposure interval of parents on wheat treated with one dose of *B. bassiana* (Bb: 1 × 10^7^ conidia/kg), two doses of spinetoram (spine1: 0.05 ppm; spine2: 0.1 ppm), their combinations (Bb + spine1; Bb + spine2) and control in seven laboratory trials carried out from 0 to 180 days after treatment

Species	Treatment	Progeny from grain infested at a given number of days after treatment								
		0	30	60	90	120	150	180	*F*	*P*
*R. dominica*	Bb	62.0 ± 2.9 Db	71.2 ± 2.6 CDb	78.2 ± 1.1 BCb	85.2 ± 3.0 ABb	92.2 ± 3.0 ABb	98.2 ± 2.0 Ab	102.2 ± 2.1 Ab	20.9	< 0.01
	Spine1	50.9 ± 3.1 Eb	56.2 ± 2.7 DEbc	63.2 ± 2.0 CDbc	69.2 ± 2.6 BCDbc	72.9 ± 2.1 ABCc	80.3 ± 2.0 ABc	86.2 ± 1.1 Abc	16.3	< 0.01
	Spine2	42.0 ± 3.9 Fb	48.6 ± 2.6 Ec	53.4 ± 1.8 DEc	60.2 ± 2.6 CDc	63.9 ± 3.2 BCc	70.3 ± 2.9 ABc	74.2 ± 3.0 Ac	48.4	< 0.01
	Bb + spine1	26.2 ± 2.8 Ec	31.2 ± 2.6 CDd	36.1 ± 2.0 CDd	40.9 ± 2.0 BCd	47.2 ± 2.3 ABd	49.2 ± 3.0 ABd	58.2 ± 2.0 Ad	48.6	< 0.01
	Bb + spine2	18.1 ± 3.4 Fc	21.7 ± 2.6 Ee	30.2 ± 2.0 Dd	35.2 ± 2.0 Cd	40.2 ± 2.0 Bd	46.2 ± 3.0 ABd	53.2 ± 3.0 Ad	130.0	< 0.01
	Control	128.4 ± 2.8 Da	133.8 ± 2.1 CDa	141.2 ± 1.0 BCa	144.9 ± 3.0 Ba	157.2 ± 1.0 ABa	151.2 ± 2.0 ABa	155.2 ± 3.0 Aa	16.3	< 0.01
	*F*	42.5	69.1	75.6	79.1	95.7	93.7	91.9		
	*P*	< 0.01	< 0.01	< 0.01	< 0.01	< 0.01	< 0.01	< 0.01		
*S. granarius*	Bb	71.2 ± 3.1 Cb	78.7 ± 3.2 Cb	89.9 ± 2.8 Bb	95.3 ± 1.1 Bb	102.1 ± 1.9 ABb	109.4 ± 2.2 Ab	115.8 ± 2.6 Ab	33.6	< 0.01
	Spine1	57.2 ± 1.5 Ec	65.7 ± 2.4 DEc	72.2 ± 3.3 CDc	78.4 ± 2.7 BCDc	83.2 ± 3.3 ABCc	89.8 ± 2.7 ABc	95.2 ± 1.0 Ac	18.8	< 0.01
	Spine2	42.0 ± 2.6 Ed	49.3 ± 1.7 DEd	57.2 ± 1.5 CDd	64.4 ± 2.0 BCd	67.2 ± 1.9 BCd	73.1 ± 2.9 ABd	84.3 ± 2.6 Ad	29.4	< 0.01
	Bb + spine1	33.2 ± 1.0 De	38.3 ± 1.9 De	45.0 ± 0.9 Ce	53.5 ± 0.9 Be	62.3 ± 1.2 ABd	69.7 ± 2.0 Ade	70.2 ± 2.9 Ae	63.9	< 0.01
	Bb + spine2	28.0 ± 1.0 Df	32.5 ± 1.1 Df	38.3 ± 1.9 Cf	44.3 ± 2.4 Cf	52.9 ± 1.4 Be	60.6 ± 1.2 ABe	65.5 ± 1.1 Ae	79.2	< 0.01
	Control	129.5 ± 2.2 Da	140.5 ± 2.0 CDa	151.2 ± 3.3 BCa	148.3 ± 3.5 ABCa	158.6 ± 2.9 ABa	137.8 ± 2.0 Aa	155.1 ± 2.4 Aa	15	< 0.01
	*F*	208.0	215.0	184.0	189.0	135.0	69.7	108.0		
	*P*	< 0.01	< 0.01	< 0.01	< 0.01	< 0.01	< 0.01	< 0.01		
*T. castaneum*	Bb	52.1 ± 2.1 Bb	61.6 ± 2.6 Bb	69.6 ± 3.4 Bb	76.0 ± 3.1 Ab	79.4 ± 3.5 Ab	84.8 ± 1.5 Ab	91.1 ± 2.6 Ab	16.9	< 0.01
	Spine1	41.5 ± 2.6 Dc	47.7 ± 1.8 CDc	51.6 ± 2.0 BCc	55.2 ± 1.8 ABc	59.3 ± 2.8 Ac	65.1 ± 2.9 Ac	72.1 ± 3.0 Ac	27.9	< 0.01
	Spine2	27.4 ± 0.9 Ed	33.3 ± 1.0 DEd	37.2 ± 1.3 CDd	42.2 ± 2.6 BCd	49.2 ± 1.4 ABd	55.8 ± 1.7 Ac	61.2 ± 3.3 Ac	30.1	< 0.01
	Bb + spine1	18.6 ± 1.5 De	25.2 ± 0.9 Dde	29.3 ± 0.9 CDe	36.4 ± 1.4 BCd	41.3 ± 2.4 ABe	43.3 ± 2.3 ABd	49.3 ± 1.7 Ad	22.1	< 0.01
	Bb + spine2	12.2 ± 0.6 Ee	18.8 ± 0.9 Df	24.0 ± 0.9 CDf	29.3 ± 0.9 BCe	35.1 ± 1.1 ABe	38.1 ± 1.2 ABd	44.9 ± 1.9 Ad	24.0	< 0.01
	Control	103.3 ± 1.9 Ca	114.7 ± 2.3 Ca	109.2 ± 1.7 BCa	121.0 ± 2.8 Ba	125.2 ± 3.2 ABa	125.6 ± 3.2 ABa	134.4 ± 3.0 Aa	3.35	< 0.01
	*F*	219.0	254.0	243.0	168.0	132.0	151.0	103.0		
	*P*	< 0.01	< 0.01	< 0.01	< 0.01	< 0.01	< 0.01	< 0.01		
*T. granarium*	Bb	141.3 ± 3.0 Eb	148.9 ± 4.5 DEb	153.4 ± 3.2 CDb	182.8 ± 4.8 BCb	171.2 ± 2.8 ABb	172.3 ± 2.0 ABb	183.9 ± 3.5 Ab	24.0	< 0.01
	Spine1	109.3 ± 4.0 Ec	119.0 ± 4.6 DEc	131.0 ± 2.2 CDc	161.8 ± 3.6 BCc	148.8 ± 3.5 ABCb	154.5 ± 4.5 ABc	161.4 ± 2.6 Ac	16.7	< 0.01
	Spine2	101.5 ± 2.2 Dc	110.0 ± 3.4 CDc	120.4 ± 4.5 BCc	127.1 ± 4.5 ABCc	138.5 ± 2.2 ABc	146.1 ± 4.0 Ac	151.6 ± 3.6 Ac	14.6	< 0.01
	Bb + spine1	81.9 ± 3.5 Dd	92.0 ± 4.0 CDd	89.8 ± 3.4 BCDd	108.3 ± 3.5 BCd	119.6 ± 3.5 ABd	126.6 ± 3.8 ABd	130.8 ± 2.2 Ad	12.2	< 0.01
	Bb + spine2	63.2 ± 3.1 De	77.1 ± 4.5 CDe	85.2 ± 3.9 BCd	94.6 ± 2.3 ABCd	104.3 ± 4.6 ABe	109.2 ± 2.6 ABe	120.4 ± 3.4 Ad	13.2	< 0.01
	Control	235.1 ± 2.5 Da	241.6 ± 3.5 CDa	226.7 ± 12.8 BCa	237.8 ± 3.0 ABCa	244.2 ± 2.8 ABa	245.5 ± 4. Aa	229.5 ± 5.7 Aa	13.9	< 0.01
	*F*	119.0	130.0	98.4	96.3	128.0	107.0	125.0		
	*P*	< 0.01	< 0.01	< 0.01	< 0.01	< 0.01	< 0.01	< 0.01		

For each species, within each treatment, means followed by the same uppercase letter are not significantly different; *df* = 6, 62, Tukey–Kramer (HSD) test at *P* = 0.05. For each species, within each trial, means followed by the same lowercase letter are not significantly different; *df* = 5, 53, Tukey–Kramer (HSD) test at *P* = 0.05

### Mortality in field trials

For *R. dominica*, all main effects and the associated interaction were significant (Table 10). At the 30th day of storage period, the combined application of *B. bassiana* plus the higher dose of spinetoram caused maximum mortality (i.e., 72.3%) while reduction in mortality was observed according the time of storage (Table 11). At the end of storage period, no treatment was able to cause 50% mortality. Mortalities in treatments were significantly higher compared to controls at all storage periods. Also, adult mortalities on wheat treated with *B. bassiana* plus the higher dose of spinetoram were significantly higher to single treatments between 60 and 180 days of storage.

**Table 10:** ANOVA parameters of main effects and associated interactions for mortalities of *R. dominica*, *S. granarius*, *T. castaneum*, and *T. granarium* adults in field persistence trials (total *df* = 323 for all species)

Species	Source	*df*	*F*	*P*
*R. dominica*	Treatment	5	1616.0	< 0.01
	Storage period	5	244.7	< 0.01
	Treatment × storage period	25	11.2	< 0.01
*S. granarius*	Treatment	5	1965.3	< 0.01
	Storage period	5	252.1	< 0.01
	Treatment × storage period	25	15.8	< 0.01
*T. castaneum*	Treatment	5	1849.5	< 0.01
	Storage period	5	236.9	< 0.01
	Treatment × storage period	25	13.6	< 0.01
*T. granarium*	Treatment	5	2098.8	< 0.01
	Storage period	5	293.6	< 0.01
	Treatment × storage period	25	12.4	< 0.01

**Table 11:** Mean mortality (% ± SE) of *R. dominica*, *S. granarius*, *T. castaneum*, and *T. granarium* adults exposed for 14 days on wheat treated with one dose of *B. bassiana* (Bb: 1 × 10^9^ conidia/kg wheat), two doses of spinetoram (spine1: 0.75 ppm; spine2: 1.25 ppm), their combinations (Bb + spine1; Bb + spine2), and control in six field trials carried out from 30 to 180 days after treatment

Species	Treatment	Trials at a given number of days after treatment							
		30	60	90	120	150	180	*F*	*P*
*R. dominica*	Bb	27.3 ± 1.6 Ac	23.4 ± 1.5 ABd	20.1 ± 1.4 Bc	17.1 ± 0.9 BCd	14.3 ± 1.0 CDd	10.8 ± 1.2 Dd	21.6	< 0.01
	Spine1	40.4 ± 2.4 Ab	37.0 ± 3.3 ABc	32.3 ± 1.3 ABCb	28.1 ± 1.5 BCc	25.7 ± 1.4 Cc	18.7 ± 1.2 Dc	18.5	< 0.01
	Spine2	52.9 ± 4.3 Aab	47.7 ± 2.9 ABbc	43.2 ± 1.9 ABb	39.6 ± 1.6 BCb	33.7 ± 1.5 CDbc	27.7 ± 2.0 Db	15.9	< 0.01
	Bb + spine1	65.1 ± 3.7 Aa	60.2 ± 4.6 ABb	52.2 ± 1.9 ABCb	47.7 ± 2.2 BCDab	42.8 ± 2.1 CDab	38.1 ± 1.4 Da	13.9	< 0.01
	Bb + spine2	72.3 ± 2.4 Aa	68.7 ± 2.7 Ba	59.4 ± 1.5 Ca	52.7 ± 2.1 CDa	47.5 ± 2.0 DEa	41.7 ± 2.1 Ea	70.5	< 0.01
	Control	6.6 ± 1.3 Ad	4.3 ± 0.6 Ae	6.6 ± 1.5 Ad	5.2 ± 0.5 Ae	3.5 ± 0.5 Ae	4.2 ± 0.5 Ae	2.1	0.09
	*F*	103.0	184.0	97.6	240.0	202.0	132.0		
	*P*	< 0.01	< 0.01	< 0.01	< 0.01	< 0.01	< 0.01		
*S. granarius*	Bb	24.6 ± 1.7 Ac	20.5 ± 1.5 ABc	17.9 ± 1.1 BCd	14.6 ± 0.8 CDd	12.7 ± 0.6 DEd	9.9 ± 0.9 Ed	23.0	< 0.01
	Spine1	37.3 ± 2.5 Ab	34.2 ± 1.4 Ab	29.1 ± 1.9 ABc	25.3 ± 1.6 BCc	21.3 ± 1.0 CDc	17.0 ± 1.3 Dc	21.9	< 0.01
	Spine2	50.0 ± 2.5 Aab	33.6 ± 1.3 ABb	41.1 ± 1.5 ABCb	37.9 ± 1.1 BCb	32.1 ± 1.1 CDb	26.1 ± 0.8 Db	12.9	< 0.01
	Bb + spine1	61.2 ± 3.9 Aa	45.0 ± 4.3 ABab	52.1 ± 2.0 Bab	46.2 ± 1.6 BCab	43.2 ± 1.5 BCab	35.0 ± 1.2 Cab	11.6	< 0.01
	Bb + spine2	67.1 ± 2.3 Aa	57.8 ± 3.1 ABa	56.5 ± 1.6 ABCa	49.9 ± 2.7 BCa	47.4 ± 1.6 Ca	37.6 ± 0.7 Da	19.8	< 0.01
	Control	5.9 ± 1.2 Ad	4.5 ± 1.0 Ad	5.8 ± 0.8 Ae	6.4 ± 0.8 Ae	3.6 ± 0.9 Ae	4.7 ± 0.7 Ae	2.0	0.25
	*F*	89.4	101.0	162.0	155.0	127.0	120.0		
	*P*	< 0.01	< 0.01	< 0.01	< 0.01	< 0.01	< 0.01		
*T. castaneum*	Bb	20.1 ± 1.6 Ad	16.5 ± 2.0 ABc	14.4 ± 1.1 ABd	11.0 ± 1.0 BCd	8.9 ± 0.8 Cd	7.7 ± 0.4 Cd	14.3	< 0.01
	Spine1	33.2 ± 2.5 Ac	29.5 ± 1.7 ABb	25.3 ± 1.7 ABc	22.9 ± 1.3 BCc	18.0 ± 0.9 Cc	13.4 ± 0.9 Dc	25.2	< 0.01
	Spine2	45.2 ± 4.3 Abc	41.9 ± 2.7 ABab	36.3 ± 1.9 ABCb	33.1 ± 1.7 BCb	29.4 ± 0.8 CDb	25.2 ± 0.8 Db	12.2	< 0.01
	Bb + spine1	56.5 ± 4.2 Aab	52.5 ± 2.0 Aa	48.3 ± 2.0 ABab	42.4 ± 1.8 BCab	38.4 ± 1.4 CDab	34.0 ± 0.7 Da	18.4	< 0.01
	Bb + spine2	63.8 ± 2.9 Aa	57.3 ± 2.9 ABa	52.7 ± 1.2 BCa	47.5 ± 2.0 CDa	42.7 ± 1.6 Da	35.1 ± 0.7 Ea	29.3	< 0.01
	Control	4.4 ± 0.7 Ae	6.1 ± 0.5 Ad	4.9 ± 0.7 Ae	5.1 ± 0.5 Ae	6.2 ± 0.8 Ae	4.9 ± 0.8 Ae	1.4	0.35
	*F*	132.0	101.0	151.0	162.0	152.0	143.0		
	*P*	< 0.01	< 0.01	< 0.01	< 0.01	< 0.01	< 0.01		
*T. granarium*	Bb	19.0 ± 2.6 Ad	15.3 ± 2.2 ABd	13.9 ± 0.6 ABd	9.5 ± 0.5 BCd	7.3 ± 0.4 Cd	6.7 ± 0.7 Cd	12.8	< 0.01
	Spine1	27.5 ± 2.6 Ac	24.7 ± 0.9 ABc	20.5 ± 0.8 BCc	16.3 ± 1.0 CDc	14.3 ± 0.4 Dc	12.3 ± 1.0 Dc	24.8	< 0.01
	Spine2	38.6 ± 2.3 Ab	35.9 ± 2.4 Ab	32.3 ± 1.3 ABb	27.3 ± 1.2 BCb	24.9 ± 0.3 CDb	20.1 ± 0.9 Db	25.8	< 0.01
	Bb + spine1	52.1 ± 1.6 Aa	46.2 ± 1.6 Aab	41.9 ± 2.1 BCa	36.0 ± 1.4 CDa	33.5 ± 0.9 DEa	31.3 ± 0.6 Ea	32.5	< 0.01
	Bb + spine2	57.1 ± 3.5 Aa	52.8 ± 1.7 Aa	45.1 ± 2.2 ABa	40.9 ± 1.4 BCa	38.1 ± 1.1 Ca	34.7 ± 0.9 Da	20.9	< 0.01
	Control	5.2 ± 1.0 Ae	6.7 ± 0.9 Ae	6.4 ± 0.7 Ae	5.1 ± 0.4 Ae	4.3 ± 0.3 Ae	4.0 ± 0.4 Ae	2.8	0.03
	*F*	69.0	80.5	180.0	248.0	487.9	167.0		
	*P*	< 0.01	< 0.01	< 0.01	< 0.01	< 0.01	< 0.01		

For each species, within each treatment, means followed by the same uppercase letter are not significantly different; in all cases *df* = 5, 53 Tukey’s HSD test at *P* = 0.05. For each species, within each trial, means followed by the same lowercase letter are not significantly different; in all cases *df* = 5, 53 Tukey’s HSD test at *P* = 0.05. Where no letters exist, no significant differences were recorded

Regarding *S. granarius*, all main effects and the associated interaction were significant (Table 10). The overall mortality did not exceed 67.1%, a value that was noticed when *B. bassiana* plus the higher dose of spinetoram were applied on wheat after 30 days of storage (Table 11). This combination provided significantly higher mortalities than the single treatments > 0 days of storage. All treatments resulted to mortalities < 38% after 180 days of storage.

Concerning *T. castaneum*, all main effects and the associated interaction were significant (Table 10). The overall mortality ranged between 20.1 and 63.8% after 30 days of storage that was progressively decreased afterwards leading to a range from 7.7 to 35.1% (Table 11). No treatment provided mortality > 50% after 120 days. Despite the fact that mortalities remained average to low, the combination of *B. bassiana* plus the higher dose of spinetoram killed significantly higher numbers of adults than treatments alone continuously after 90 days of storage.

As far as *T. granarium* is concerned, all main effects and the associated interaction were significant (Table 10). After 30 days of storage, the lowest mortality was 19.0% while the highest was 57.1% on wheat treated with *B. bassiana* alone and *B. bassiana* plus the highest dose of spinetoram respectively (Table 11). The same combination was the only one that provided the death of 52.8% of the exposed adults while all other treatments provided adult mortalities between 15.3 and 46.2% after 60 days of storage. The storage period progressively reduced mortalities reaching after 180 days a maximum value of 34.7% on wheat treated with *B. bassiana* plus the higher dose of spinetoram.

## Discussion

The combination of *B. bassiana* with the higher dose of spinetoram caused higher mortality rates and greater progeny inhibition than all the other tested formulations, both in laboratory and in field experiments, against *R. dominica*, *S. granarius*, *T. castaneum*, and *T. granarium* adults. On the basis of the results of the current study, *R. dominica* was the most susceptible species followed by *S. granarius*, *T. castaneum*, and *T. granarium*, in laboratory and field experiments when *B. bassiana* and spinetoram (both doses) were applied alone or combined. Interestingly, when Wakil et al. (2022) tested one dose of *B. bassiana* (1 × 10^7^ conidia/kg), two doses of fipronil (0.05, 0.1 ppm), and their combinations, as well as when Wakil et al. (2021d) tested seven entomopathogenic fungi isolates against the same species, the susceptibility ranking was the same. Although spinetoram exhibits elevated insecticidal activity against several species of stored-product insects (Vassilakos et al. 2012, 2015; Saglam et al. 2013; Vassilakos and Athanassiou 2015; Ksoura et al. 2021), previous studies revealed that it also affects various life history parameters of field pests, e.g., the diamondback moth, *Plutella xylostella* (L.) (Lepidoptera: Plutellidae), under sublethal doses of 0.047 mg/l (Tamilselvan et al. 2021). Even when *P. xylostella* was exposed at 0.072 mg spinetoram/l, the adult emergence, the rate of pupation, and the pupal weight got reduced in the two successive generations of this species. The F_1_ generation fecundity and the intrinsic rate of increase were also decreased.

The increase of temperature increased mortality and decreased progeny production of all insect pests at all tested treatments. Similarly, at commodities treated with *B. bassiana* against *R. dominica*, *S. granarius*, *T. castaneum*, and *T. granarium* adults, the increase of temperature caused the death to more individuals (Wakil et al. 2022). Temperature plays an important role in the development of the entomopathogenic fungi and therefore in their insecticidal performance (Michalaki et al. 2006; Wakil et al. 2011; Athanassiou et al. 2017). More specifically, between 25 and 32 °C, *B. bassiana* exhibits the fastest germination, while at 30 °C displays the fastest growth (James et al. 1998). Different species of entomopathogenic fungi require different temperature ranges for their optimal growth. For instance, *I. fumosorosea* exhibited elevated insecticidal activity against *R. dominica* at 25 °C, rather than at 20 or 30 °C (Riasat et al. 2013). In contrast, Vassilakos et al. (2006) observed higher insecticidal properties of *B. bassiana* as wheat protectant at 26 °C compared to 30 °C against adults of *R. dominica* and *S. oryzae*. This could be attributed to the different *B. bassiana* isolates, as they exhibit different conidia germination and relative growth, depending on their geographic region (Uma Devi et al. 2005; Wakil et al. 2021d). Apart from the insecticidal performance of the entomopathogenic fungi, temperature can influence the effectiveness of spinetoram. For example, more maize weevils, *Sitophilus zeamais* Motschulsky (Coleoptera: Curculionidae), adults were killed on maize treated with spinetoram as the temperature increased from 20 to 30 °C (Yılmaz et al. 2020). Similarly, it caused the death of more *R. dominica*, *S. oryzae*, and *T. confusum* adults as temperature increased from 20 to 25 and 30 °C (Vassilakos and Athanassiou 2013). The same pattern was documented in the case of *P. truncatus*, *R. dominica*, *S. oryzae*, and *T. confusum* adults, when exposed to spinetoram alone or in combinations with spinosad (Athanassiou and Kavallieratos 2014). Likewise spinetoram, another active ingredient that belongs to the spinosyn family, spinosad, caused higher mortality rates to *R. dominica*, *S. oryzae*, *T. confusum*, and *P. truncatus* adults at 30 °C than at 25 and 20 °C (Athanassiou et al. 2008b). In the current study, the increase of mortality rates caused by spinetoram could be attributed to the fact that high temperatures tend to increase the metabolic activities, and consequently the stress of the insects after the contact with the insecticide (Athanassiou and Kavallieratos 2014).

Concerning the progeny production, the F1 individuals were fewer at all tested treatments for all tested species and temperatures, than the control group. The lowest emergence of offspring was recorded at 30 °C, followed by 25 and 20 °C. This could be explained by the high mortality levels at 30 °C, resulting to fewer parental individuals that produced fewer eggs. Despite the elevated efficacy of the tested combinations, the total suppression of progeny production was not achieved. Similarly, the progeny production of *P. truncatus* individuals exposed on wheat treated with 0.01 and 0.1 ppm chlorantraniliprole increased with an increase of temperature from 20 to 25 and 30 °C (Boukouvala and Kavallieratos 2021). The inhibition of progeny is also documented by a plethora of other insecticides like chlorfenapyr, pirimiphos-methyl, etofenprox, and spinosad compared to the control assays (Pozidi Metaxa and Athanassiou 2012; Athanassiou and Kavallieratos 2014; Boukouvala and Kavallieratos 2022). Since the mode of action of spinetoram is the disruption of *γ*-aminobutyric acid and nicotinic acetylecholine receptors (Depalo et al. 2016), the nervous system is compromised (Millar and Gotti 2009). Low progeny can be attributed to paralysis and neuromuscular fatigue (Salgado et al. 1998; Fahmy and Dahi 2009), which may result to reproductive inability and ovulation of the parental adults.

The combination of *B. bassiana* and the higher spinetoram dose as grain protectants provided elevated protection after 30 days but after 180 days, the protection was moderate in both laboratory and field trials, against all tested species. Furthermore, the combination of *B. bassiana* and the lower dose of spinetoram led to lower mortality values than the previous combination, but higher than the single treatments of *B. bassiana* and spinetoram at both doses, for all species tested in the laboratory or field and during the residual tests. Therefore, the combination of *B. bassiana* and spinetoram resulted in additive toxicity effects against all coleopterans. Previous reports have revealed that the combinational use of *B. bassiana* with synthetic insecticides improves their overall insecticidal performance. For instance, thiamethoxam in combination with *B. bassiana* (1.5 × 10^8^ conidia/kg wheat) and the DE SilicoSec (200 ppm) effectively reduced *R. dominica* individuals (Wakil et al. 2012). Similarly, Wakil and Schmitt (2015) documented that the efficacy of the combination *B. bassiana* plus DE plus imidacloprid, as wheat protectants, almost suppressed *C. ferrugineus*, *R. dominica*, *T. castaneum*, and *L. paeta* adults over a period of 6 months. Recently, Wakil et al. (2022) found that *B. brassiana* + fipronil resulted to elevated mortality against *S. granarius*, *T. castaneum*, and *T. granarium*. When spinetoram was tested alone, mortality levels were low after 180 days in both laboratory and field trials. Recently, Ksoura et al. (2021) reported the stability and the residual efficacy of spinetoram after 5 months on maize and wheat against *S. oryzae* and *R. dominica* adults. The authors found that spinetoram at 0.1 ppm resulted to 100% mortality to *R. dominica* adults and 25.4% to *S. oryzae* adults after 5 months of storage, 14 days post-exposure. In contrast, at least for *R. dominica* adults, after 150 days of storage, mortality rates were 37.9 and 33.7% for laboratory and field trials respectively, 14 days post-exposure. Therefore, the residual efficacy of spinetoram is a species- and/or strain-dependent phenomenon, as it has been previously observed for other insecticides (Zettler and Arthur 1997; Vayias et al. 2006; Kavallieratos et al. 2007; Rossi et al. 2010). Further research is needed to clarify this issue.

The long-lasting elevated properties of insecticides can be attributed to different modes of actions (Athanassiou and Kavallieratos 2014) as well as to the stability of each insecticide through time (Wakil and Schmitt 2015; Ksoura et al. 2021). However, the prolonged effectiveness of several synthetic insecticides may not be considered desirable since many have long-term impact to the environment, non-target organisms, and human health (Ansari et al. 2014; Okunola et al. 2014; Berjawi et al. 2020). Hence, low-toxicity insecticides can be exploited in combination with biological control agents to reduce the recommended label doses further.

In conclusion, the current study provides useful data concerning the combination of the entomopathogenic fungus *B. bassiana* with the bacterial insecticide spinetoram against several major stored-product insects under different abiotic factors. We showed that its exploitation in combination with a biological control agent is feasible for a prolonged storage period, an issue that triggers its application as grain protectant at low doses. The climate crisis and the global warming (Sognnaes et al. 2021; Yang et al. 2022; NASA 2022) favor the dispersal of noxious insect species including stored-product insects (Athanassiou et al. 2019; Papanikolaou et al. 2019; Kim et al. 2020; Singano et al. 2020). Therefore, temperature becomes an important component of studies that evaluate the performance of insecticides, alone or in combination with other agents, for the management of harmful insects occurring in storage facilities. Spinetoram exhibits residual efficacy against several stored-product insects, as it has been revealed in the current study and in Ksoura et al. (2021). Considering also the fact that it lacks cross-resistance to other classes of insecticides (Watson et al. 2010; Sparks et al. 2012), it becomes a suitable active ingredient for IPM and insecticide-resistance programs (Sparks et al. 2012; Lira et al. 2020). Further evaluation of this insecticide in combination with other species/strains of entomopathogenic fungi and natural products may provide additional knowledge towards the concept of protection of stored-products with low-risk tools.

## Data Availability

The datasets used and/or analyzed during the current study are available from the corresponding author on reasonable request.
